# Global Sensory Impairment Independently Predicts Decreased Social Function Over Time

**DOI:** 10.1093/geroni/igaa057.1335

**Published:** 2020-12-16

**Authors:** Anna Huang, Kristen Wroblewski, Ashwin Kotwal, Linda Waite, Martha McClintock, Jayant Pinto

**Affiliations:** 1 University of Chicago Medical Center, Chicago, Illinois, United States; 2 University of Chicago, Chicago, Illinois, United States; 3 University of California, San Francisco School of Medicine, San Francisco, California, United States

## Abstract

The classical senses (vision, hearing, touch, taste, and smell) play a key role in social function by allowing interaction and communication. We assessed whether sensory impairment across all 5 modalities (global sensory impairment [GSI]) was associated with social function in older adults. Sensory function was measured in 3,005 home-dwelling older U.S. adults at baseline in the National Social Life, Health, and Aging Project and GSI, a validated measure, was calculated. Social network size and kin composition, number of close friends, and social engagement were assessed at baseline and 5- and 10-year follow-up. Ordinal logistic regression and mixed effects ordinal logistic regression analyzed cross-sectional and longitudinal relationships respectively, controlling for demographics, physical/mental health, disability, and cognitive function (at baseline). Adults with worse GSI had smaller networks (β=-0.159, p=0.021), fewer close friends (β=-0.262, p=0.003) and lower engagement (β=-0.252, p=0.006) at baseline, relationships that persisted at 5 and 10 year follow-up. Men, older people, African-Americans, and those with less education, fewer assets, poor mental health, worse cognitive function, and more disability had worse GSI. Men and those with fewer assets, worse cognitive function, and less education had smaller networks and lower engagement. African-American and Hispanic individuals had smaller networks and fewer close friends, but more engagement. Older respondents also had more engagement. In summary, GSI independently predicts smaller social networks, fewer close friends, and lower social engagement over time, suggesting that sensory decline results in decreased social function. Thus, rehabilitating multisensory impairment may be a strategy to enhance social function as people age.

